# Efficiency of Human Papillomavirus (HPV) Testing in Cervical Cancer Screening and Vaccination: A Review

**DOI:** 10.7759/cureus.102126

**Published:** 2026-01-23

**Authors:** Shirin Dasgupta

**Affiliations:** 1 Dr. B. C. Roy Multi-Specialty Medical Research Centre, Indian Institute of Technology Kharagpur, Kharagpur, IND

**Keywords:** cervical cancer screening, hpv dna, hpv screening, hpv vaccination, prevention

## Abstract

Cervical cancer is one of the leading causes of female mortality in the world. However, since it has a long premalignant stage, cervical cancers can be prevented by early diagnosis or by regular screening. Over the years, conventional cytology has served as an excellent tool for diagnosis and early detection of cervical dysplastic lesions. However, it had a few drawbacks, which were overcome by the introduction of liquid-based cytology (LBC). LBC provided a relatively better efficiency. However, LBC necessitates high-end laboratory infrastructure, which is unavailable in resource-poor settings.

Human papillomavirus (HPV) being the main etiology for causing cervical cancer, theoretically, cervical cancer can be prevented if vaccination against HPV is provided at an early age. Also, HPV testing, in contrast to cytology, does not depend on morphology for interpretation and is only based on the identification of HPV DNA or other viral markers. HPV detection thus provided less room for false positivity than cytology.

In this review, the author will focus on the importance of HPV testing as a potential primary cervical cancer screening modality. Also, this review will discuss the advent of HPV vaccination with its role in cervical cancer prevention.

## Introduction and background

Cervical cancer constitutes the third most common cancer amongst women globally [1]. While developed countries continue to expand the screening methods and vaccination programs, females in developing countries and resource-poor settings face gross disparities in accessing them. Tragically, the low and middle-income countries face a bulk of deaths due to cervical cancer. Approximately 311,000 patients succumb to cervical cancer annually, with nearly 90% of them from low- and middle-income countries [1]. Nevertheless, advanced technologies have been developed to permit a more rapid and cost-effective cervical cancer screening [1].

Human papillomavirus (HPV) is the primary etiology of most of the cervical cancers [2]. Essentially, it is very much possible to prevent cervical cancer. The measures include primary prevention with HPV vaccination, secondary prevention through cervical screening with detection of precancerous lesions and treatment, as well as tertiary prevention via early cancer diagnosis and treatment [2]. However, access to these effective preventive measures remains insufficient in many resource-poor settings.

Although cytology-based screening programs were implemented by developed countries from the mid-20th century, the main decline in cervical cancer occurrence was observed only when a coverage over 70% was enabled by national call-and-recall programs [3]. Continued screening is essential as cytology has a poor sensitivity (~53%) as per a meta-analysis from North America and Europe [4]. The finding that persistent HPV infection in cervical cancer led to the development of HPV detection tests and HPV vaccines. Compared to cytology, HPV tests were found to have more sensitivity (96.1% vs. 53.0%) but less specificity (90.7% vs. 96.3%) [4]. Thus, HPV testing is more appropriate as a screening test [5]. However, in the beginning, there was an unwillingness to consider it as a primary screening measure. Initially, the American Society for Colposcopy and Cervical Pathology (ASCCP) put forward HPV testing as a measure for triage for atypical squamous cells of undetermined significance (ASC-US) cytology results [5]. Eventually, the notion of co-testing with cytology appeared, and finally, HPV testing was accepted as the primary screening modality for cervical cancer [6,7].

When George N. Papanicolaou and H. F. Traut invented the Pap smear test in the 1940s, cervical cytology constituted a strong and less complex technique for cervical cancer screening [8]. Pap test constitutes the exfoliation of cervical cells with the help of a brush. These cells are then fixed in alcohol, stained, studied under a microscope, and then morphologically inferred. Although the Pap smear is used as a primary screening technique on a large scale, it has a few limitations. The procedure of staining the slides in a conventional Pap test requires a significant amount of time (20-30 minutes) [8]. Cervical cytology is reported on subjective interpretation of morphological changes in the exfoliated cells of the cervix and, therefore, may be associated with errors in interpretation. Moreover, cytology needs to be repeated multiple times, which mostly yield negative results (the most serious limitation), leading to fatigue that habitually causes screening errors in interpretation and missed lesions. The advent of liquid-based cytology (LBC) as opposed to the conventional smear has improved the efficiency in processing the cervical samples in screening programs [9]. However, LBC requires high-end laboratory infrastructure, which is not available in resource-poor settings.

HPV testing, in contrast to cytology-based screening programs, does not depend on morphology for interpretation and is solely based on the detection of HPV mRNA, HPV DNA, or other viral markers. HPV DNA testing has proven to be 30%-40% more sensitive than cytology but is less (5%-10%) specific in detecting high-grade lesions [10]. In the past two decades, several countries have included HPV testing as a vital part of clinical guidelines for cervical cancer screening, triage, and post-treatment follow-up [11].

The etiological connection between persistent high-risk HPV infection and cervical cancer has created new methods in cervical cancer prevention. The HPV vaccine is a potent tool in preventing cervical cancer. Combined with cervical cancer screening programs, the HPV vaccine can nearly eradicate death due to cervical cancer. Nevertheless, the success of this outstanding public health measure can only be assessed if the vaccines can be provided to the population who are at most risk; that is, unscreened women. Providing vaccination to a well-screened population would hardly have any impact on improving the mortality of cervical cancer mortality, thus it will be a prominent public health disappointment [12].

This review will focus on the importance of HPV DNA testing as a possible primary cervical cancer screening modality, as well as the advent of HPV vaccination with its implications on cervical cancer prevention.

## Review

Methodology

To compile this review, articles under the headings “HPV screening”, “HPV vaccination”, “Cervical cancer screening,” and “Cervical cancer prevention” have been extensively searched in PubMed/Medline and Google Scholar. The author has gone through around 100 publications, including original research and review articles pertaining to this topic.

HPV as a screening tool for cervical cancer

For decades, cervical Pap cytology has been practiced as a screening method for cervical cancer and its precursors in most developed countries, either as individual events or as opportunistic programs. A young HPV vaccinated woman will carry a significantly lower risk of suffering from precancerous lesions of the cervix over a prolonged period of time, extending for a decade or even longer after becoming sexually active. Therefore, exhaustive Pap cytology screening of already vaccinated women is unnecessary, which wastes resources and provides just minimum additional advantage [13].

A relatively feasible and sensible strategy for screening of cervical cancer is to utilize the highly sensitive HPV DNA testing as the first-line screening modality and the highly specific Pap smear test as the second-line screening modality in women who turn out to be HPV DNA (HPV/Pap triage). Despite being accurate and efficient in identifying the active high-grade cervical lesions, the HPV/Pap triage paradigm is yet to be implemented for screening because of the dearth of long-term follow-up studies regarding the safety of prolonged screening intervals. Nevertheless, an analysis of pooled European studies has established that a negative HPV DNA test carries a lower risk of interval precancer in women than that of a negative Pap test. Essentially, a negative HPV DNA test is more reassuring for the patient (than a negative Pap test) that she carries low risk while she waits for the next screening opportunity [14].

If HPV DNA testing is used in place of Pap cytology as the primary screen, it is expected that in any group of HPV-positive cases, the prevalence of cytological abnormalities will be significantly higher, about 20% or more. It can be seen that cytology will have its highest PPV and hence greatest clinical utility provided lesion prevalence is maintained at a high level. Under such circumstances, it is expected that the screening work conditions will considerably improve, since the cytotechnician will keep in mind that the Pap smears to be reported are *flagged *because they originate from HPV-positive women and are more likely to be premalignant or malignant lesions. Eventually, this will result in less tiresome work and identify lesions more accurately [15]. Furthermore, if solely HPV testing is used as the primary screening modality, there will be a significant reduction in Pap smear reporting workload for cytology personnel. This, apart from reducing costs, will also increase the thoroughness and attention with which each smear will be attended [13]. Yet another major benefit of applying HPV DNA testing as the primary screening modality is that extended screening intervals can be implemented, which will be cost-effective compared to a cytology-centered screening program. [13].

HPV screening is still considered superior to cytology screening even though there is a fair chance of missing HPV-negative cancers. Moreover, HPV genotyping cannot effectively differentiate the different steps in cancer development, such as a long-lasting transient or a persistent infection and a prevalent precancerous lesion [16]. Notably, a tiny percentage of cervical cancers are truly HPV negative [16]. Prevention of these HPV negative cancers cannot be attained by HPV vaccination or HPV screening. Hence, the colposcopy and biopsy should be undertaken promptly for clinically suspicious cases, for early diagnosis and reducing the possibility of missing diagnosis. Whether or not the cervical cancer patients who are HPV negative require a customized treatment plan is food for advanced research, but the primary HPV screening should definitely be continued [16].

Meta-analyses have summarized many studies and compared the cross-sectional accuracy of HPV testing to that of cytology screening. These results showed greater sensitivity but lower specificity of HPV DNA testing compared to cytology [7]. Increased referral to colposcopy with HPV testing in comparison to cytology is related to its lower specificity.

High-grade cervical intraepithelial neoplasia (CIN) is detected by HPV testing earlier than cytology, allowing more time for intervention before invasion. This is expected to permit longer screening intervals with a lower or similar risk of cancer. In a very large cohort of women routinely screened by HPV and cytology co-testing [17], the five-year cumulative incidence of cancer was 3.8 per 100,000 after a negative HPV test, 3.2 per 100,000 after a negative co-test, and 7.5 per 100,000 after a negative cytology.

HPV-based screening carries certain limitations, which include high cost, requirement for high-end laboratory infrastructure, and a long processing time. Primary HPV screening should be available on a large scale in low/ middle income countries. Also, point-of-care (POC) and affordable HPV tests need to be available to allow single visit approach [18].

Currently, the global market possesses about 250 distinctive commercial HPV tests, but only a few of them are clinically validated [8]. The efficacy of an HPV test is dependent upon several factors, such as sample collection technique, nucleic acid extraction procedure, primers, and internal controls. The selected assay should ideally be type-specific as well as HPV genome region-specific (L1, E1/ E2, and E6/E7). A newly chosen HPV assay needs to be similar to a gold standard HPV test, which has already been validated by a randomized controlled trial (RCT) [19,20].

The efficiency of primary HPV screening has been investigated by many well-designed clinical trials. Pooled data from more than 1.2 million women have confirmed the effectiveness of primary HPV screening [18].

CIN2+ and CIN3+ yielded higher detection rates of 42% and 33%, respectively, by using co-testing rather than only cytology. The respective specificity (CIN2+ and CIN3+) of co-testing was 6% and 8% less than cytology alone. Combining cytology with HPV screening increased sensitivity by just 5% and 2% for CIN2+ and CIN3+, respectively, when compared to sole HPV testing [11].

In another study, the sensitivity of the HPV test when assessed for CIN1 and CIN2 was better than cytology [21]; however, when it was assessed for CIN3 and higher grade lesions (CIN3+), the sensitivity of the HPV test was comparable to that of the conventional Pap. Also, the positive predictive value and specificity of the HPV test were lower compared to the Pap smear. Pap test and HPV test, when combined, showed higher sensitivity than only cytology, but less specificity [22].

A study assessing the relative role of cytology and HPV testing in CIN and cervical cancer detection established that more preinvasive and invasive lesions were detected only by the HPV component when compared with cytology. Merely, 3.5% of pre-cancers and 5.9% of cancers were cytology positive and HPV negative. Therefore, the cytology test contributed to just five cases per million cases per year to the sensitivity of the combined test [23].

The World Health Organization (WHO) recommends that each country use a high-performance HPV test to screen at least 70% of women aged 35 to 45 years by 2030 for the global elimination of cervical cancer [18].

The introduction of the HPV test for cervical cancer screening was not uniformly accepted: some authors specified that noncompliance with protocols, as well as opportunistic screening, may widen the already existing disparities since HPV is costlier than the Pap test. Additionally, prolonged intervals can disrupt women’s habits and thus have unfavorable effects on the screening coverage [24].

Screening for cervical cancer is a potentially lifesaving intervention, with several approaches, including liquid-based cytology, HPV molecular testing, and combination methods such as sequential triage or parallel co-testing. It is essential to maximize screening efficacy while simultaneously diminishing overtreatment.

However, the screening modalities are yet to be perfect. Although HPV testing carries more sensitivity than LBC, its decreased specificity and higher frequency of false-positive cases increase the possibility for needless and possibly injurious consequences [25].

It is important to consider how HPV screening can be implemented in primary cervical cancer screening in the best possible way as it becomes cheaper and more available. Multicenter studies provide evidence that the HPV-based primary screening provides greater prevention from invasive cervical carcinomas compared to LBC. Studies have supported the theory that every five-yearly HPV screening could cut down unnecessary colposcopies as well as biopsy procedures when compared with regular LBC. Significant evidence also suggests that negative high-risk HPV tests provide longer and greater reassurance of a reduced risk of cellular abnormality compared to negative cytology results. Against this backdrop, the authors have suggested that high-risk HPV primary screening may be implemented as an alternative to U.S. cytology-based cervical cancer screening methods. HPV testing can accelerate the efficiency of primary cervical cancer screening and also lessen the potential damage due to over-screening [26].

Of late, HPV testing is being studied by many investigators as an isolated primary screening modality. In a large U.S.-based study of primary HPV screening, the HPV test was found to be as effective or more effective than cytology alone as a primary cervical cancer screening tool. In 2015, the ASCCP published interim guidelines for the use of the FDA-approved HPV test for primary cervical cancer screening, declaring it may be considered an alternative in women 25 years and older [1]. It has been predicted that primary HPV screening may become the standard primary cervical cancer screening tool within the next few years [1]. The biggest challenges with HPV testing are the cost, requirement for laboratory processing, and high turnaround time. The development of low-cost, self-swabbing, and quick HPV testing should expedite rapid screening on a large scale [1].

Can HPV vaccination prevent cervical cancer?

HPV vaccination is a potent tool for cervical cancer prevention. This vaccine, along with cervical cancer screening programs, can almost always prevent cancer mortality.

Since 2006, the HPV vaccine has been available in the United States. It is considered safe and is indicated for the prevention of genital warts as well as cervical, vulvar, vaginal, anal, and penile cancer. The newest version of the Gardasil 9 vaccine can prevent up to 90% of cervical cancers [27]. Although the vaccines were initially designed for women aged 9 to 26, their use was later expanded to include women up to age 45. The Advisory Committee on Immunization Practices (ACIP) recommends administering routine vaccination at ages 11 or 12. Only two doses of the vaccine are required if vaccinated before age 15; but if vaccination is given at 15 years or later, three doses are required [28].

Gardasil was the first prophylactic HPV vaccine to be approved in the United States in 2006, and shortly after, in 2009, Cervarix (GlaxoSmithKline, Rixensart, Belgium) was also approved. Both these vaccines target two of the 12 oncogenic HPV types (HPVs 16 and 18), responsible for the bulk of cervical cancer cases worldwide [29]. The freshly launched Gardasil 9 targets another five oncogenic HPV types, thus contributing to another 20% of cervical cancer cases globally, and will have an even greater effect on disease prevalence and screening performances [30].

Mortality reductions can be observed only when there is high vaccine uptake, for example, the 80% coverage target promoted by Healthy People 2020 [30]. Furthermore, studies have shown noticeable wide inconsistency in HPV vaccine uptake [31], signifying there might be disparities in the incidence of cervical cancer if vaccination is focused among the already screened or higher socioeconomic strata.

It has been reported that HPV vaccine uptake is significantly lower among Asian, Black, and Latina women in the United States compared with White women [12]. Some studies report higher coverage among Hispanic, Asian, and Black teenagers compared with White teenagers, due to a steeper rise in uptake [32].

Post-licensure monitoring of the impact of HPV vaccines is crucial to monitor the progress made toward cervical cancer elimination and also to recognize areas where further progress can be made to fast-track the achievement of this vital public health goal. Over the last few years, a significant amount of evidence has discovered substantial benefits of HPV vaccination in preventing cervical infections and precancers at the individual-level (i.e., direct effectiveness), as well as in diminishing the population-level burden of disease (i.e., overall effectiveness). It can be stated that currently, the effectiveness of the HPV vaccines in preventing cervical cancer is just beginning to materialize since there is a prolonged latency period for the development of invasive disease [33].

Vaccine efficacy refers to the direct protection that an individual attains by a complete vaccination course; this can be witnessed in ideal conditions of RCTs directed in highly limited population cohorts. It is considered that vaccine efficacy represents an inherent vaccine property (i.e., biologic efficacy). Placebo-controlled prelicensure clinical trials displayed a high efficacy (>95%) of all the HPV vaccines against the vaccine-type HPV infections of the cervix as well as precancerous lesions [34]. This almost-perfect efficacy signifies how potent the HPV vaccines are and the enormous health benefits that can be attained from them in daily clinical settings [33].

Postlicensure monitoring studies can be utilized to measure vaccines using real-world data as well as observational study designs. These can evaluate direct as well as overall effectiveness. Direct effectiveness denotes the individual-level protective benefits, and it measures the fraction of disease or infection that is prevented among the vaccinated patients in real-world clinical scenarios. This is measured by relating the incidence of the vaccine-related outcomes in the vaccinated as well as the unvaccinated individuals. On the other hand, overall effectiveness deals with the advantage of vaccines at the population level by estimating both direct and indirect effects. Indirect effects refer to the benefits that unvaccinated individuals may gain due to a portion of the cohort being vaccinated [33].

A systematic review estimated the effectiveness of the tetravalent vaccine for the prevention of vaccine-type cervical infections to be 36%-89%, depending upon the age of immunization and number of doses received. An efficiency of 12%-84% against high-grade cervical lesions was reported [35]. Comparable findings about the effectiveness for bivalent have also been detected. For instance, the effectiveness of the bivalent HPV vaccine in preventing HPV16/18 infections was reported to be 94% among women aged 18-26 years in a Spanish study [36]. In England, 82% effectiveness was observed among women vaccinated before the age of 15 years [37]. As expected, efficacy has consistently been shown to be higher in the younger population, due to the higher probability of being vaccinated at younger ages before natural exposure to HPV.

Many researchers have also put forward the effectiveness of HPV vaccines in lowering the problem of infections as well as precancerous lesions of the cervix at the population level. A systematic review and meta-analysis condensed the data after analyzing 36 available reports from 14 high-income nations, including up to nine years of follow-up after vaccination [38]. The meta-analyses reported reductions in HPV16/18 infections of 83% among females aged 13-19 years, 66% among those aged 20-24 years, and 37% among those aged 25-29 years. These drops were similar in studies that inspected bivalent (three studies) and tetravalent vaccines (11 studies). Reductions in precancerous lesions in the screened population were reported to be 51% among females aged 15-19 years and 31% among females aged 20-24 years. Both HPV infections and other cervical lesions showed higher decline rates in nations with a wider coverage for HPV vaccination [33].

Though HPV vaccination introduction had a great acceptance rate, and probably will prove to be the definitive solution in primary cervical cancer prevention, it has to be kept in mind that this vaccination is exclusively a prophylactic one, i.e., it does not have any therapeutic effect in treating already established cervical infections. Thus, the already infected women hardly benefit [39]. It has questionable potency in eliminating already established infections [40]. Moreover, due to parental refusal or suboptimal healthcare delivery, a proportion of young women who can actually benefit from vaccination are not immunized. So, it is essential to continue screening for cervical cancer even in the age of HPV vaccination [13].

Precancerous and cancerous lesions screening, along with vaccination-driven primary prevention, is instrumental in diminishing the frequency of HPV-related ailments and death effectively [41]. Currently, three prophylactic licensed HPV vaccines are available: Gardasil is a quadrivalent HPV vaccine (4vHPV) targeting HPV16, HPV18, HPV6, and HPV11; Gardasil 9 is a nonavalent HPV vaccine (9vHPV) targeting HPV16, HPV18, HPV31, HPV33, HPV6, HPV11, HPV52, HPV58, and HPV45; and Cervarix is a bivalent HPV vaccine (2vHPV) targeting HPV16 and HPV18 [16]. These vaccines are effective in reducing up to 70%-90% of cervical cancer-related deaths.

Ideally, HPV vaccination should deliver an enriched protective immunity, protecting from all the high-risk HPV types and also other possible and probable carcinogenic types. Presently, HPV vaccines are built on virus-like particles (VLPs). The VLPs are non-infectious because they lack viral DNA and are collected from recombinant HPV capsid proteins. VLPs can initiate different types of type-specific neutralizing antibodies, which bind to the built-in virus particles and deactivate the virus by stopping its uptake via the target cell [16].

HPV vaccines not only protect against HPV infections but also provide immunity to any succeeding viral infection by inducing high and long-lasting neutralizing antibody titers. The HPV vaccination induced immune responses are considerably more potent than those in natural HPV infections. However, these vaccines have limited capacity in eliminating pre-existing infections and produce type-restricted protection. It is required that HPV carry a wider range of VLP variants for expansive protection. Ongoing research exposes that the antibody titers which are produced by Gardasil, Gardasil 9, and Cervarix and vaccination can linger for at least 9.9, 5, and 10 years, respectively, provided women in the age group 9-15 years receive a three-dose vaccination regimen [16]. Various studies conducted reveal that HPV16 and HPV18 antibody levels post 2vHPV vaccination lasted for at least 9.4 years, which were significantly higher than the natural infection levels [42]. 4vHPV vaccination provided stable HPV16 antibody levels for at least nine years [43]. Gardasil 9 vaccination of three doses provided HPV antibodies, which were present through five years, and seropositivity ranged from 77.5% to 100% [44].

A meta-analysis of the quadrivalent HPV (4vHPV) vaccine showed that, after vaccination, infections with HPV6, HPV11, HPV16, and HPV18, as well as genital warts, were reduced by up to 90% compared with the unvaccinated cohort. Also, the occurrence of low-grade cervical cytological abnormalities, as well as high-grade histologically diagnosed cervical abnormalities, showed a dip by 45% and 85%, respectively. [35]. Although the prophylactic HPV vaccinations were primarily designed considering a three-dose vaccination regimen, research studies have stated that even one or two doses of the 2vHPV vaccine showed high efficacy compared to the three-dose schedule [45]. Decreasing the number of doses not only leads to reductions in overall cost, which is a problem (especially in low-income countries), but it also increases the loyalty to the program [46].

Early evidence of the impact of HPV vaccination on the incidence of cervical precancerous lesions has been demonstrated in developed countries with established HPV vaccination programs, such as Australia [47], Scotland [48], and the United States.

Also, a few studies have examined the effectiveness of HPV vaccination on invasive cervical cancer. Results from long-term passive follow-up studies of two large HPV vaccination trial cohorts (65,656 woman-years) and unvaccinated control cohorts (124,245 woman-years) conducted in Finland from 2007 to 2015 discovered 100% efficacy if HPV vaccine among invasive cervical cancer cases [49]. Also, a large-scale study of HPV vaccine effectiveness in Sweden observed a reduction of 63% incidence of invasive cervical cancer among vaccinated women compared to the unvaccinated women, with a higher reduction of 88% among women who were vaccinated before 17 years of age [50].

Among all HPV-related cancers, cervical cancer is most strongly HPV associated, as the cancer development is almost always related to persistent high-risk HPV infection. This implies that, theoretically, HPV vaccination (along with screening and appropriate treatment of precancerous cervical lesions) can eradicate cervical cancer (defined by the WHO as lower than 4 new cases per 100 000 women per year) [51].

Existing HPV vaccines target HPV18 and HPV16, which contribute to about 70% of total cervical cancers [52]. Around 90% of cervical cancers can be prevented by including 5 added oncogenic HPV types (HPV33, HPV31, HPV52, HPV45, and HPV58) in the HPV vaccine [53]. The bivalent HPV (2vHPV) vaccine provides considerable cross-protection against the non-vaccine HPV types 31, 33, and 45 [54]. Added protection against HPV types 6 and 11 is provided by the quadrivalent vaccine (4vHPV), which causes >90% of anogenital warts. The latest vaccine added, 9vHPV, was introduced in 2015, and it provided protection against infection with another five high-risk types (HPV33, HPV31, HPV52, HPV58, and HPV45). WHO reported that, by the end of 2019, more than 100 countries had incorporated HPV vaccination into their national immunization programs [33].

Because progression from HPV infection to cervical cancer and its subsequent clinical diagnosis is a prolonged process, clinical vaccine trials and postlicensure observational studies have used cervical precancerous lesions as proxy endpoints for cervical cancer [51]. Fourteen years postlicensure of the HPV vaccine, a population-based study showed that the risk of cervical cancer among girls who were vaccinated before 17 years of age was 88% less than among unvaccinated girls [50].

A cohort study conducted nationwide showed that HPV vaccination lowers cervical cancer incidence by 86% and 68% among individuals vaccinated at or before 16 years of age or 17-19 years, respectively. This scenario was not very perfect for women who were vaccinated at 20-30 years of age, where the incidence rate of cancer was somewhat similar to that of unvaccinated women. These findings provide evidence that early age vaccination is more effective for cervical cancer prevention. Thus, when vaccination is combined with regular screening, it should hypothetically be possible to eliminate cervical cancer [51].

The astounding international success of the HPV vaccine is already apparent [38], and nations with a wider coverage of HPV vaccination are already seeing a decline in the rates of precancerous lesions and, of late, abnormal cervical cells. A cohort study [50] found that the positive predictive value of cytology was significantly diminished for girls who were vaccinated, with sharper dips in girls vaccinated at younger ages.

Trials that led to the approval of Gardasil and Cervarix showed that these vaccines are almost 100% effective in providing immunity against persistent cervical infection and dysplasia caused by HPV types 16 and 18 [1].

## Conclusions

Regarding cervical cancer prevention, there are two important considerations: one is attaining the technique to attain a higher vaccination coverage, and whether cervical cancer screening should be continued once a high vaccination coverage is attained. Based on this review, it can be inferred that the population who are not screened is the high-risk population for developing cervical cancer. HPV testing allows earlier detection of high-grade cervical precancerous lesions than cytology, thus providing greater lead time before invasion. This is expected to authorize longer screening intervals with a lower or similar risk of cancer. However, since HPV screening is more expensive than the conventional cytology screening test (Pap smear), the introduction of HPV testing as the primary screening method may suffer from social inequality. The identification of HPV as the main causative agent of cervical cancer paved the path for the development of HPV vaccines. Although continuing observational studies enlighten us about the long-term effect of the HPV vaccine on rates of cervical cancer, it will take several years before we have any evidence about its efficacy.

The introduction of diagnostic HPV tests, which provide more accuracy and sensitivity than primary cytology testing, has lately caused a paradigm shift. Whether HPV vaccination actually prevents cervical cancer development in the long run needs to be studied further with a large sample pool and for a long duration. Also, the percentage of the underscreened population has to be reduced so that the efficacy of such a ground-breaking vaccination can be accurately highlighted.
